# Recruitment of multiple stakeholders to health services research: Lessons from the front lines

**DOI:** 10.1186/1472-6963-10-123

**Published:** 2010-05-13

**Authors:** Michelle E Kho, Ellen Rawski, Julie Makarski, Melissa C Brouwers

**Affiliations:** 1 Department of Clinical Epidemiology and Biostatistics, McMaster University, Henderson Site, 60 (G) Wing, 2nd Floor, 711 Concession Street, Hamilton, ON L8V 1C3, Canada; 2 Department of Oncology, McMaster University, Henderson Site, 60 (G) Wing, 2nd Floor, 711 Concession Street, Hamilton, ON L8V 1C3, Canada; 3 Program in Evidence-Based Care, Cancer Care Ontario, McMaster University, Henderson Site, 60 (G) Wing, 2nd Floor, 711 Concession Street, Hamilton, ON L8V 1C3, Canada

## Abstract

**Background:**

Self-administered surveys are an essential methodological tool for health services and knowledge translation research, and engaging end-users of the research is critical. However, few documented accounts of the efforts invested in recruitment of multiple different stakeholders to one health services research study exist. Here, we highlight the challenges of recruiting key stakeholders (policy-makers, clinicians, guideline developers) to a Canadian Institutes of Health Research (CIHR) funded health services research (HSR) study aimed to develop an updated and refined version of a guideline appraisal tool, the AGREE.

**Methods:**

Using evidence-based methods of recruitment, our goal was to recruit 192 individuals: 80 international guideline developers, 80 Canadian clinicians and 32 Canadian policy/decision-makers. We calculated the participation rate and the recruitment efficiency.

**Results:**

We mailed 873 invitation letters. Of 838 approached, our participation rate was 29%(240) and recruitment efficiency, 19%(156). One policy-maker manager did not allow policy staff to participate in the study.

**Conclusions:**

Based on the results from this study, we suggest that future studies aiming to engage similar stakeholders in HSR over sample by at least 5 times to achieve their target sample size and allow for participant withdrawals. We need continued efforts to communicate the value of research between researchers and end-users of research (policy-makers, clinicians, and other researchers), integration of participatory research strategies, and promotion of the value of end-user involvement in research. Future research to understand methods of improving recruitment efficiency and engaging key stakeholders in HSR is warranted.

## Background

Expectations for well-designed self-administered surveys are high[1] and results can only be drawn and generalized based on the quantity, quality and representativeness of information returned[2]. Therefore, achieving a high participation rate is a significant precursor to ensuring the validity of survey results and minimizing the risk of bias. Studies show a trend towards decreased participation in survey research [3]. Thus, we need methods to facilitate participation. A Cochrane systematic review and meta-analysis identified several key methods to enhance response rates to postal questionnaires, including a more versus less interesting questionnaire, recorded delivery, and receipt of a monetary incentive[2]. In contrast to research aimed at improving response rates, however, there are few documented accounts of the efforts invested in participant recruitment and the resultant participation rates for this investment. The purpose of this short report is to outline our experiences recruiting practice guideline developers/researchers, clinicians and policy-makers to a Canadian Institutes of Health Research (CIHR) funded health services research (HSR) study. The object of this study was the Appraisal of Guidelines Research and Evaluation (AGREE) Instrument, a tool used to evaluate the quality of practice guidelines (PGs) reporting [4].

## Methods

### Identification of target participants and sampling strategy

Following an *a-priori *sample size calculation for our primary outcome, our total recruitment target was 192: 80 Canadian clinicians, (oncology, cardiovascular, and critical care), 80 international guideline developers/researchers, and 32 Canadian policy/decision-makers. Based on previous specialist response rates to the 2004 Canadian National Physician survey, we expected to approach 4 physicians for every physician we needed to recruit[5]. We also applied the same oversampling rate to the guideline developers/researchers and policy-makers. We identified potential participants using membership lists from professional associations, known research/clinician collaborations, and professional entities found on the Internet (see Table 1). From this population, we invited a random sample of clinicians and guideline developers/researchers, with e-mail addresses, to participate. As we had fewer candidates, we invited all identified policy-makers to participate. Informed consent was implied with the return of completed survey materials. The Hamilton Health Sciences/McMaster University Faculty of Health Sciences Research Ethics Board approved this study.

**Table 1:** Recruitment data sources.


**Participant Type**	**Guideline Developers**	**Clinicians (Oncologists, Intensivists, Cardiologists)**	**Policy/Decision-Makers**

Data sources	-AGREE partners -Canadian Medical Association Guidelines Infobase -Canadian Partnership Against Cancer Corporation -Conference on Guideline Standardization (COGS) participants -Grading of Recommendations, Assessment, Development, and Evaluation (GRADE) Working group -Guidelines International Network	Publicly-available lists from the websites of the following provincial Colleges of Physicians and Surgeons: -Alberta -British Columbia Manitoba -New Brunswick -Newfoundland and Labrador -Nova Scotia -Ontario -Prince Edward Island	-Canadian Agency for Drugs and Technologies in Health -Cancer Care Ontario (Committee to Evaluate Drugs, Clinical Program Heads, Clinical Council) -Canadian Pharmacists' Association -Health Canada (Chronic and Continuing Care Division, Health Products and Food Branch, Pharmaceuticals Management Strategies, Therapeutic Effectiveness and Policy Bureau) -Ontario Health Technology Advisory Committee

At the time of our study, the following provinces or territories did not have publicly available physician lists: Saskatchewan, Nunavut, and the Northwest Territories. The province of Quebec did not have a list of physicians by specialty (only by physician name). The Yukon Territory limited the use of their physician data for informational purposes only.

### Description of self-administered questionnaire

Our research protocol involved four parts: i) reading a PG; ii) assessing the PG using either the AGREE Instrument and the Global Rating Scale (Condition 1) or the Global Rating Scale alone (Condition 2); iii) completing a survey of perceptions of the usefulness of the instrument(s) from (2); and iv) completion of a short demographic section. The PGs included 10 documents of 3 clinical areas (4 oncology, 4 cardiovascular, 2 critical care), and all PGs were 50 pages or less. We randomized participants to either Condition 1 (134 items total) or Condition 2 (41 items). For clinicians, we stratified randomization to their corresponding area of expertise (e.g., oncologists randomly assigned to condition 1 or condition 2, and randomized to 1 of 4 oncology PGs). We randomized policy-makers to oncology PGs alone, because of a smaller pool of participants. Finally, we randomly allocated developers/researchers to condition and guideline. Further details about the primary research protocol and survey instruments are described elsewhere[6].

From pilot testing, the estimated time to complete all three parts was no more than two hours for those in Condition 1, and approximately 1.5 hours for Condition 2. We sent the initial survey by personally addressed e-mail, which included direct electronic links to the study materials. Participants had the option of completing the survey electronically or by paper. In turn, participants could choose to submit their completed survey materials electronically via the secure online data portal http://www.vovici.com, by electronic mail word processing document, by post mail or by fax.

To inform our recruitment efforts, we used a systematic review summarizing evidence-based strategies for recruitment[2] and a narrative review of key methodological steps in survey administration[1]. We incorporated a modified Dillman approach[7] in our recruitment strategies: we pre-contacted participants via personally addressed letters on McMaster University letterhead followed by a personally addressed e-mail or individual telephone call 10 days later to ascertain their participation[2,8]. We offered participants a $100 CDN gift certificate incentive upon completion of study materials. All participants submitting data received a personalized note of thanks. For all participants with outstanding submissions, we followed up with two reminder e-mails and/or telephone calls and resent the complete study package with the second email reminder, as per our protocol. Our protocol allotted and resourced for 6.5 months to complete participant recruitment and data collection.

### Outcomes

Using a screening log, we recorded the number of eligible people and those approached to participate in the study [9]. Of those approached, we recorded the number of undeliverable letters, affirmative responses, active declines, and non-responses. We calculated the participation rate (number who agreed to participate over the total number approached)[10], and the recruitment efficiency (proportion of completed data submissions as a function of the number of letters sent)[9].

### Results

Recruitment and data collection took nearly twice as long as we anticipated. Table 2 outlines our recruitment efforts. Between June 7, 2007 and April 2, 2008 we mailed 873 invitation letters to 173 developers, 526 clinicians, and 174 policy-makers. Thirty five letters were undeliverable.

**Table 2:** Recruitment efforts.

	Developers	Clinicians			Policy/Decision-Makers	Total
			
		Oncology	Cardiology	Critical Care		
Recruitment target	80	32	32	16	32	192
**Initial invitation**						
Letters sent	173	222	245	59	174	873
Letters undeliverable	9	5	17	1	3	35

**Total approached**	**164**	**217**	**228**	**58**	**171**	**838**
Agreed to participate	93	52	29	21	45	240
Declined participation	71	165	199	37	126	598
Active decline	26	53	75	16	60	230
No response	45	112	124	21	66	368

**Participation rate**	**56.7%**	**24.1%**	**12.7%**	**36.2%**	**26.3%**	**28.6%**

**Participant follow-up**						
**Agreed to participate**	**93**	**52**	**29**	**21**	**45**	**240**
Data received	70	31	14	14	27	156
Withdrawn	4	5	1	1	8	19
Deferred	0	0	0	2	0	2
No data received	19	16	14	4	10	63

**Recruitment efficiency**	**42.7%**	**14.3%**	**6.1%**	**24.1%**	**15.8%**	**18.6%**

Oncologists included medical and radiation oncologists. Declined participation includes active declines, and no responses. Two developers, who were not on our recruitment lists, volunteered to participate in the study. One developer, who also held cardiology credentials was grouped into cardiologists. One oncologist, who was also a policy-maker, was grouped into policy/decision-makers. Participation rate = Agreed to participate/Total approached; Recruitment efficiency = Data received/Total approached.

Of 838 pre-contacted, our participation rate was 29% (240). We received data from 65% (156/240) of the individuals who agreed to participate, representing a recruitment efficiency of 19% (156/838) of the original sample invited to participate. Of those who submitted data, 95% (148) used the online data portal, 7 submitted their data by electronic mail (word processing document), and 1 submitted their data by post. No respondents returned their data via fax. Of those participating and submitting data, we actively monitored each submission for complete data. We had no missing data for the main study primary outcomes.

We followed-up with 333 reminder e-mails and 61 telephone calls. Of the reminder e-mails sent, 215 were second follow-ups and contained a complete electronic survey package as per our protocol. Developers/researchers were more likely to participate than clinicians and policy-makers. Of those initially agreeing to participate, 8% (19) actively withdrew from the study and from 26% (63) we received no data. One policy-maker manager did not allow the participation of policy staff who already gave consent, accounting for 5 of 8 policy-maker withdrawals. Of the 19 withdrawals, 26% (5) occurred before randomization, 42% (8) were allocated to Condition 1 (the longer condition), and the remaining 32% (6) were allocated to Condition 2. Of the 63 who did not submit data, 43% (27) were allocated to Condition 1 and 57% (36) to Condition 2.

## Discussion

Research productivity is dependent on timely receipt, analysis, and publication of data, which is ultimately dependent on study sample participation. The validity and generalizability of survey results are dependent on a high participation rate and representative sample. We incorporated the best available evidence to optimize our participation rates[2] and used previously reported response rate estimates to guide our recruitment efforts[5]. While the number of individuals who originally agreed to participate was 25% higher than our target sample size, we still missed our target by 17%.

Based on our experiences, and in contrast to previous research [5], we received 1 person's data for every 5 letters of invitation. Guideline developer/researcher recruitment was highest, probably reflecting their existing interests in this area. Clinician and policy-maker recruitment was more challenging. Our clinician recruitment rates were much lower than previous studies, where recruitment rates for medical oncologists, radiation oncologists, and cardiologists were 33.0%, 36.9%, and 28.4%, respectively (response rates unavailable for critical care)[5]. We found similar responses for policy-makers.

As has been found elsewhere[3,11], reasons for our low recruitment rate might include seasonality, lack of interest, limited time or lack of perceived relevance. Despite the three-fold difference in the total number of questionnaire items between Condition 1 (n = 134) and Condition 2 (n = 41), there was little impact as a function of study load; more participants who did not complete data came from Condition 2, the less demanding study condition. Of particular interest in our case, we learned that some policy-makers were actually dissuaded by their superiors from participating. Although this may be an isolated incident, this is an interesting finding nonetheless and suggests further fostering the much needed collaboration between the research and policy/decision-making entities. Further, lack of anonymity may have dissuaded others from participating in the study.

Health services research often relies on the participation of different stakeholder groups "in the field" to yield findings that can be useful and relevant to improve the system. Knowledge translation efforts depend on stakeholder involvement[12]. We need continued efforts to communicate the value of research between researchers and end-users of research (policy-makers, clinicians, and other researchers), integration of participatory research strategies[13], and promotion of the value of end-user involvement in research. Our research team included perspectives from each of the target groups we sought to recruit. However, given the breadth of coverage of stakeholder groups we sought to recruit (perspectives and geography) it may be that we did not include all "typical" phenotypes.

## Conclusions

Based on the results from this study, we suggest that future studies aiming to engage similar stakeholders in HSR over sample by at least 5 times to achieve their target sample size and allow for participant withdrawals. Continued use of appropriate evidence-based strategies to increase survey response rates is important, with a particular emphasis on highlighting the relevance of the study to the prospective participants and the importance of their participation. Further, we suggest ongoing dialogue about how to best engage end-users. While our recruitment strategies for physicians and policy-makers were specific to the Canadian health care system, we suggest that the underlying principles are applicable to any systematic effort at identifying a population sample. Future research to understand methods of improving recruitment efficiency and engaging key stakeholders in HSR is warranted.

## Competing interests

The authors declare that they have no competing interests.

## Authors' contributions

Conception (MEK, MCB), design (MEK, JM, ER, MCB), data acquisition (MEK, JM, ER), analysis (MEK, MCB) and interpretation of data (all). Drafting the article (MEK), critical revisions for important intellectual content (JM, ER, MCB). All authors of this paper have read and approved the final version submitted. MK had full access to all the data in the study and is the guarantor for the integrity of the data and the accuracy of the data analysis.

## Authors' Information

Melissa Brouwers is the Principal Investigator of the AGREE II Next steps project (CIHR #77822). Michelle Kho is funded by a Fellowship from the Canadian Institutes of Health Research (Clinical Research Initiative). This study was funded by the Canadian Institutes of Health Research, who had no role in the design, analysis, or interpretation of the data.

## Pre-publication history

The pre-publication history for this paper can be accessed here:

http://www.biomedcentral.com/1472-6963/10/123/prepub
